# Public-Private Partnerships and Public Health Practice in the 21st Century: Looking Back at the Experience of the Steps Program

**Published:** 2009-03-15

**Authors:** Alyssa Easton

**Affiliations:** Healthy Communities Program, Centers for Disease Control and Prevention

To address the health and financial burden of chronic disease in communities and advance public health practice in the 21st century, the Centers for Disease Control and Prevention's Steps Program, established in 2003, funds selected national organizations and communities nationwide to implement evidence- and practice-based community interventions. These interventions focus on reducing obesity, diabetes, and asthma and addressing the underlying risk factors of physical inactivity, poor nutrition, and tobacco use. Building on successes and lessons learned from the Steps Program, CDC has broadened its investment in communities through the creation of CDC's Healthy Communities Program, of which Steps is now a part. The Healthy Communities Program mobilizes communities across the nation to take local action to prevent and reduce the impact of chronic disease. Many key policy, systems, and environmental decisions that affect chronic disease rest in the hands of local decision makers.

Steps communities accelerate positive change toward health and wellness at the local level by creating a groundswell of activity across local community, school, work site, and health care settings. Public-private partnerships (ie, partnerships between the public and for-profit sectors) have become a valuable public health tool for stemming chronic disease at the community level and have played an important role in enhancing, expanding, and sustaining Steps Program activities. Given the tremendous chronic disease burden faced by local communities across the country, these nontraditional partnerships, by thinking outside the box, serve to improve community- and individual-level health and provide renewed hope for bringing about positive change.

The rise in chronic disease rates in the United States followed a century of profound cultural changes. In the 19th and early 20th centuries, people often struggled to get enough food, faced unsanitary living conditions, and engaged extensively in physical labor — notably on farms and in factories. Today, most people struggle to balance increasingly demanding work and family obligations. Hectic schedules, labor-saving technology, high-calorie convenience foods, widespread promotion of unhealthy foods and tobacco products, and the increase in sedentary behaviors have caused chronic disease rates to explode.

During the last century, chronic diseases replaced infectious diseases as the nation's greatest health threat. In 2005, chronic diseases affected more than 50% of Americans (1) and accounted for 7 of the 10 leading causes of death in the United States: heart disease, cancer, stroke, chronic lower respiratory disease, diabetes, Alzheimer's disease, and kidney disease (2). Though largely preventable, chronic diseases are among the most prevalent and costly of all health issues. In 2000, the estimated annual cost in the United States of obesity alone (including direct medical costs and indirect costs related to disability) was $117 billion (3).

Restoring healthier behaviors to people and society will require another cultural shift, one just as dramatic as the previous century's. This shift will be driven by setting-specific health promotion activities that include nontraditional partnerships. Public-private partnerships play an important role in initiating, supporting, and sustaining activities to improve the health of communities. These partnerships are the focus of this special issue.

## The Steps Program and its Private-Sector Partners in 2007

Steps communities have benefited in multiple ways from public-private partnerships. In addition to providing funds to Steps communities, private-sector partners have promoted Steps activities, shared their expertise and resources, participated on leadership teams and in community coalitions, assisted with the implementation of Steps activities, and brought to Steps communities an audience that they might otherwise not have had access to.

### Colleges and universities

Steps communities partnered with 35 universities that provided expertise in evaluation and dissemination by assisting with the development of evaluation plans, ongoing evaluation efforts, and preparation of manuscripts for publication.

### Health care organizations and health insurance companies

Steps communities partnered with 29 health care organizations that helped implement specific disease management and prevention programs. These organizations provided clinical and behavioral health program expertise that enhanced Steps activities in health care settings. Steps communities also partnered with pharmacies that disseminated information about Steps programs. For example, a Steps community partnered with a local pharmacy that strategically posted information about recognizing asthma symptoms in a campaign to increase asthma awareness. Steps' physician partners have promoted Steps interventions by referring patients to tobacco-use cessation programs and local quitlines, diabetes education and self-management training sessions, and weight management and nutrition education classes. In addition, Steps communities partnered with 20 insurance companies that make patient referrals to Steps health care activities.

### Businesses

Steps communities partnered with 45 small and large businesses that became an important asset. For example, local restaurants and taquerías were provided with culturally relevant materials and concepts developed by their local Steps program to promote healthy food and beverage consumption. The eateries began to increase the number of healthy options on their menus, posted signs that promoted healthy eating, and created a network of healthy restaurants in the community. Vending machine businesses have also partnered with Steps communities, providing more healthy snacks and beverages in their vending machines in schools, work sites, and health care and community facilities.

### Media organizations

Steps communities partnered with 42 media organizations that promoted Steps activities. These partnerships have provided newspaper, television, radio, billboard, and Internet banner advertisements that were not otherwise affordable to Steps communities. The organizations also gave Steps programs access to large, local audiences that could be targeted with specific and culturally relevant messages. To assist Steps communities with marketing strategies, these private-sector partners also provided health messaging and other marketing expertise that benefited Steps health promotion activities.

Financial support — from direct funding or in-kind support through sharing expertise, resources, or other services — has been an important benefit of the Steps Program's private-sector partnerships. Though many partnerships have continued for years, our most recent data, a snapshot of 2007, provides a sense of the types of partnerships and financial support associated with the Steps Program (Figure). In 2007, more than 80% of Steps communities had at least 1 private-sector partnership; the average was 5. Among the Steps communities that collaborated with private-sector partners, funding amounts (direct and in-kind) ranged from $25 to $140,000 per partner and from $2,500 to $360,000 per community in 2007. Financial support is important in establishing and sustaining new health promotion activities, but it is especially beneficial when private-sector partners also provide their expertise and take an active role in planning, promoting, and implementing community-based activities.

**Figure 1:**
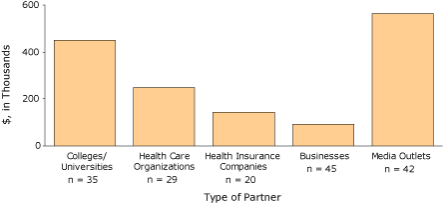
Types of private-sector partners and their financial and in-kind contributions, 2007 Steps Program

**Table:** 

## Steps Program's Private-Sector Partnerships in Community Settings

Public-private partnerships have expanded the Steps Program's ability to develop, promote, and implement successful local health promotion strategies in 4 settings: work sites, schools, health care facilities, and the community. I encourage you to read, in this issue of *Preventing Chronic Disease,* about 2 examples of Steps programs that have partnered with the private sector to improve employee wellness: Davis and colleagues discuss the impact and sustainability of a partnership between the Austin Steps Program, Austin's Capital Metropolitan Transportation Authority, and Health & Lifestyles Corporate Wellness, Inc, that decreased absenteeism and health care costs (4); Hawkins and colleagues discuss the WorkWell initiative, a program developed by the Thurston County Steps Program and the Thurston County Chamber of Commerce (5). This program assists employers that implement programs to help employees achieve a healthy weight and be physically active and recognizes local employers that implement related policy, systems, and environmental changes. The program developed an assessment tool to guide public-sector employers in identifying successful activities to support workforce health promotion.

Steps communities are also collaborating with the private sector to improve student health in schools. The Cleveland Steps Program, in collaboration with the YMCA of Greater Cleveland and the Cleveland Metropolitan School District, works with the Rite Aid Cleveland Marathon to encourage healthy habits and exercise in students. Through the "We Run This City" Youth Marathon Program, young people are taught to set and achieve goals, increasing their sense of self-efficacy and self-confidence as well as their fitness levels and endurance. A total of 283 student runners in grades 6 through 12 representing 26 different schools participated in the annual marathon's events in May 2008, more than 3 times the number of participants in the first year of the program in 2006. Most students (210) completed the final 1.2-mile portion of the marathon, and nearly 3 times as many students as from the previous year — 71, up from 23 — ran the entire 10 km event. Two students who have been in the program since its inception have worked their way up to having completed the half marathon this year. Through this program, schools have learned new ways to make physical activity fun and to engage youth, parents, and the entire community in health promotion.

In the hospital setting, the Chautauqua County Steps Program in New York, in collaboration with the local tobacco control program, partnered with hospital staff members of the Women's Christian Association Hospital to implement a policy requiring health care providers to directly address patients' tobacco use and efforts to quit. Health care providers from several disciplines were trained by wellness coordinators and the local Steps program facilitator to follow the 5 A's intervention model, a 2-minute strategy shown to increase tobacco-use cessation, with every patient (6). This intervention led to an increase in total calls to the state's quitline and in the number of smokers who have been able to quit using tobacco.

With a community-wide focus, the Salinas Steps Program in California, in collaboration with the Monterey County Health Department, partnered with Brown-Miller Communications to create the "Value It" campaign, a multifaceted presentation of health messages through outdoor murals and radio, newspaper, and television ads, which promotes the importance of personal health. This campaign contributed to a 12% increase in the number of people with healthy weight in Salinas' Latino population and was awarded 2 of the nation's highest public relations honors from the Public Relations Society of America in 2008. Another successful public-private partnership for improving health community-wide was established between the Clark County Steps Program in Washington and the National Automated Merchandising Association. The National Fit Pick Vending Program helps consumers select healthy choices from vending machines. Fit Pick uses standardized sets of nutrition guidelines based on American Heart Association recommendations for a healthy diet and is available to vending operators nationwide. The identification system is designed to be recognizable to consumers throughout the country. To support the program, the Clark County Steps Program developed step-by-step tool kits and print-ready promotional materials for vendors, work sites, schools, and communities. The nation's vending industry unveiled the new health-oriented vending program with the support of the Clark County Steps Program in April 2008.

As evidenced by diverse examples and experiences from Steps Program collaborations with private-sector partners, these relationships and the innovative activities they spark are not only the possibilities of successful approaches to improving health but also their realization. Together, these partnerships represent a 21st century public health approach — one guided not only by evidence but also by nontraditional alliances — that can begin to turn the tide on chronic disease.
